# Nutcracker syndrome: how are we cracking the nuts and whose nuts are we cracking?

**DOI:** 10.1590/S1677-5538.IBJU.2016.0517

**Published:** 2017

**Authors:** Fernando Korkes

**Affiliations:** 1 Departamento de Urologia, Hospital Israelita Albert Einstein, São Paulo, SP, Brasil

*To the editor,*

The Nutcracker phenomenon refers to the compression of the left renal vein between the superior mesenteric artery and the aorta, and is often asymptomatic. It is not uncommon, and can be found in up to 10.9%-14% of asymptomatic adults (1, 2) and 33% of children with hematuria (3). The Nutcracker syndrome (NCS) comprises symptoms and findings such as varicocele, ovarian vein syndrome, hematuria, proteinuria and flank pain.

Computed tomography and magnetic resonance imaging can demonstrate the anatomic abnormality, and Doppler ultrasonography can help to measure pressure gradient and diameter differences between the left renal vein at the hilum and at the aortomesenteric level. Phlebography might be of value when there are doubts (4). Treatments include clinical management in most cases, and weight gain might be of benefit (5). Nephrectomy, reno-caval re-implantation or shunts (open, laparoscopic or robotic), external stents (open, laparoscopic or robotic) and endovascular venous stents have also been reported (4, 6, 7). Since surgical alternatives are invasive and outcomes are not outstanding (8), endovascular stents have emerged with the appeal of a less invasive procedure. However, some statements have to be reinforced.

First, as procedures seems less invasive, there is an apparent increment in the number of indications. We have been seeing a significant increase in the number of cases of tomographic diagnosis of Nutcracker phenomenon. More than that, a significant number of patients have been treated with venous stents, even in cases of doubtful indications.

The Nutcracker syndrome is a benign and in most cases self-limited condition, that occurs almost commonly in young patients (resolve with time) (5). Treatment is reserved only for the severe cases.

Additionally, venous stents are currently poorly understood. Distinctively from the large acquired experience with arterial stenting, there is relatively little experience and no long term studies with venous stenting. A crucial difference between arterial and chronic venous disease is that the latter seldom poses a threat to life. The venous system might be particularly prone to some severe complications. Fibromuscular hyperplasia, which can lead to vascular occlusion, seems to be more common in veins than in arteries. Proximal embolization can occur, as had the experience with vena cava filter demonstrated (9).

Experience with inferior vena cava filters have demonstrated that there is a significant risk of long term complications including IVC thrombosis, perforation, penetration of adjacent viscera, device migration and deep vein thrombosis. These complications get more common according to dwell time (9). For these reason, temporary devices have been developed (9). Additionally, current stents are not ideally developed for the venous system (9). In the last years, an impressive increase in the number of complications of these procedures have been reported. We have reviewed the current literature and counted 816 cases of NCS reported. Of those, 354 were managed clinically (43%); 160 were managed through open, laparoscopic or robotic surgery (20%); and there are 224 reports of endovascular management of NCS (27%). From 2000 to 2005, 12 cases of stent placement for NCS were reported; from 2006 to 2010, 23 cases; and from 2011 to 2016, 189 cases have been reported. But impressively, there were also 21 reports of these venous stents used to treat NCS that have migrated, either immediately after placement or up to 12 months after surgery. Of the total of patients with NCS treated with stents reported in the literature, stent migration occurred in 9% of the cases. Stent migration is relatively common after endovascular stenting. In one of the largest series of endovascular stents, 75 young patients received venous stents for this benign condition. After a short term follow-up, stent migrated in five cases (7%). And unfortunately, there were not any anatomic or stent related factor that could predict this severe complication.

The authors of this study have concluded that venous stent migration in patients with NCS is much more common than believed (11). In other small series, migration occurred in 17%-20% of cases (8, 12). And almost all of these cases, migration occurred to the vena cava or to the heart, in many cases with serious complications requiring open heart surgery and even valvar replacement (11, 13, 14).

Moreover, long term venous stents patency is uncertain with stents. For this reason, experts in the field strongly recommend the maintenance of antiplatelet agents or anticoagulation following endovascular treatment of Nutcracker syndrome (15). But considering that we are treating young patients, mainly women with 20-40 years, future pregnancies can become risky (16) and lifetime anticoagulation can bring additional concerns.

It is very important to stress some points. Nutcracker syndrome is not always an easy diagnosis. Most patients should be treated conservatively because spontaneous remission does occur. It has been observed that 75% of patients younger than 18 years old will have complete resolution of hematuria within two years of presentation (4). Similarly, asymptomatic patients with incidental findings of Nutcracker phenomenom should be managed conservatively, as the natural history of such findings is not well delineated (1). Intervention shall only be indicated in severe lesions when there are disabling symptoms that do not respond to conservative management. When treatment is to be considered, care should be taken, and the best technique should be discussed with the patient or his/her parents. And venous stents don’t seem to be a good alternative for this young population of patients with this generally benign disease. We have to take care about whose nuts are we cracking and how we are cracking those nuts.

**Figure 1:**
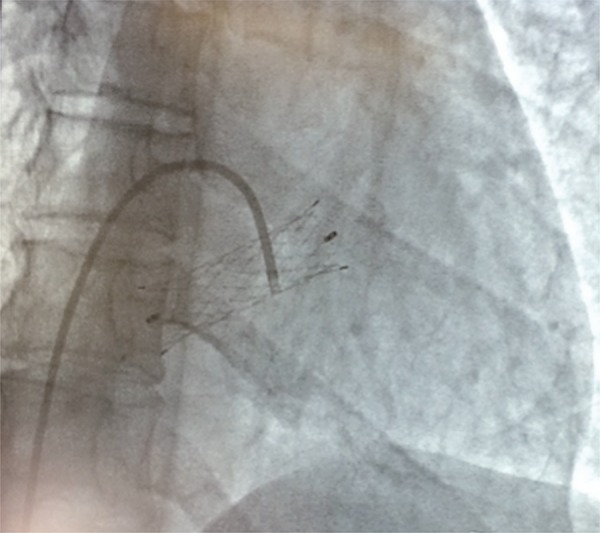
Atrial migration of a venous stent rendered this 35-year-old woman with a severe valvar insufficiency.
